# Role of the tumor microenvironment in malignant melanoma organoids during the development and metastasis of tumors

**DOI:** 10.3389/fcell.2023.1166916

**Published:** 2023-04-19

**Authors:** Siyu Zhou, Jinghan Lu, Shiyang Liu, Jiaqi Shao, Zhanwei Liu, Jianjun Li, Wan’an Xiao

**Affiliations:** Department of Orthopedics, Shengjing Hospital of China Medical University, Shenyang, Liaoning, China

**Keywords:** patient-derived organoids (PDO), extracellular matrix (ECM), cancer-associated fibroblasts (CAFs), tumor microenviroment (TME), melanoma, immunetherapy

## Abstract

Malignant melanoma (MM) is the most metastatic and aggressive form of skin cancer, and carries a high risk of death. Immune-checkpoint inhibitor therapy and molecular-targeted therapy can prolong the survival of patients with advanced MM significantly. However, the low response rate and inevitable drug resistance prevent further improvements in efficacy, which is closely related to the tumor microenvironment (TME). The TME refers to the tumor stroma, including fibroblasts, keratinocytes, immune cells, soluble molecules, and extracellular matrix (ECM). The dynamic interaction between the TME and tumor cells is very important for the growth, local invasion, and metastatic spread of tumor cells. A patient-derived organoid (PDO) model involves isolation of tumor tissue from patients with MM and culturing it *in vitro* in a three-dimensional pattern. Compared with traditional cultivation methods, the PDO model preserves the heterogeneity of the tissue structure of MM and demonstrates the interaction between MM cells and the TME. It can reproduce the characteristics of proliferation, migration, and invasion of MM cells, and better simulate the structural function of MM *in vivo*. This review explores the role of each TME component in development of the PDO model. This review will provide a reference for research on the drug screening and targeted treatment using PDOs, particularly for the immunotherapy of MM.

## Introduction

Malignant melanoma (MM; also known as “melanoma” and “cutaneous melanoma”) is the most lethal type of skin malignancy worldwide. It is very difficult to treat due to its high malignancy, aggressiveness, and short duration of disease, which makes MM prone to metastasis (Millet et al., 2017; Lodde et al., 2020). Metastasis to the liver occurs in 14–29% and metastasis to the brain occurs in 12–20% of patients with metastatic cutaneous melanoma, with low 5-year survival rate and an extremely poor prognosis (Rosenberg and Finger, 2008). Some studies have shown that MM incidence is increasing gradually worldwide, thereby posing a serious threat to human health and increasing the economic burden upon patients (Hartman and Lin, 2019; Ahmed et al., 2020).

MM development involves a pivotal process of progressive transformation of melanocytes from antioxidants to pro-oxidants, which results in DNA damage and leads to cancer (Obrador et al., 2019). This intricate process is influenced by a range of etiologies, including UV radiation, genetic factors, among others (Trucco et al., 2019; Abdo et al., 2020). Moreover, dysregulated activation of signaling pathways such as MAPK and PI3K/PTEN/AKT pathways, due to genetic alterations, such as BRAF and KIT mutations, further promote MM proliferation (Strashilov and Yordanov, 2021). Therefore, investigating the expression and regulation of key genes in MM development is important to understand the cause of MM and the prognosis of patients suffering from MM.

Traditional treatments for MM are surgery, chemotherapy, and radiotherapy, but the prognosis for patients with distant metastases is poor due to the low sensitivity of MM cells to radiotherapy and chemotherapy. Targeted therapy and immunotherapy have prolonged the survival and improved the quality of life of patients, especially for patients with unresectable stage-III or stage-IV MM. Immunotherapies such as immune-checkpoint inhibitors (ICIs; e.g., programmed cell death protein (PD)-1), lytic immunotherapy, and cellular immunotherapy can be used in combination to increase the efficacy of treatment. Targeted therapies include inhibitors of BRAF proto-oncogene, serine/threonine kinase and mitogen-activated protein kinase. Dual-targeted therapy and targeted therapy combined with immunotherapy have achieved better efficacy than use of these agents alone (Flaherty et al., 2010; Kim et al., 2013; McArthur et al., 2014; Latimer et al., 2015; Gutiérrez-Castañeda et al., 2020). There have been great advances in immunotherapy and targeted therapies, but their widespread use in clinical practice is limited by their severe side-effects, susceptibility to drug resistance, and expense. Therefore, identifying potential targets for efficacious treatment of MM, screening for sensitive drugs, and conducting research on drug combinations and drug resistance are important challenges for the medical community.

The heterogeneity of human cancers presents a challenge in the screening of targeted drugs and ICIs for cancer treatment (Gerlinger et al., 2012; Jamal-Hanjani et al., 2017). Traditional two-dimensional (2D) cell cultures in *ex vivo* experiments fail to represent the morphology and heterogeneity of tumors accurately.

In contrast, organoids (a type of three-dimensional (3D) cell culture) have gained popularity as models for drug screening since establishment of the first organoid system by Clevers and colleagues in 2009. Since then, organoids have become a highly sought-after topic for research in 3D models (Sato et al., 2009). Organoids can recreate tumor morphology *in vitro*, retain the components of tumor cells, and allow observation of dynamic changes in the tumor, all in a shorter culture time.

**FIGURE 1:**
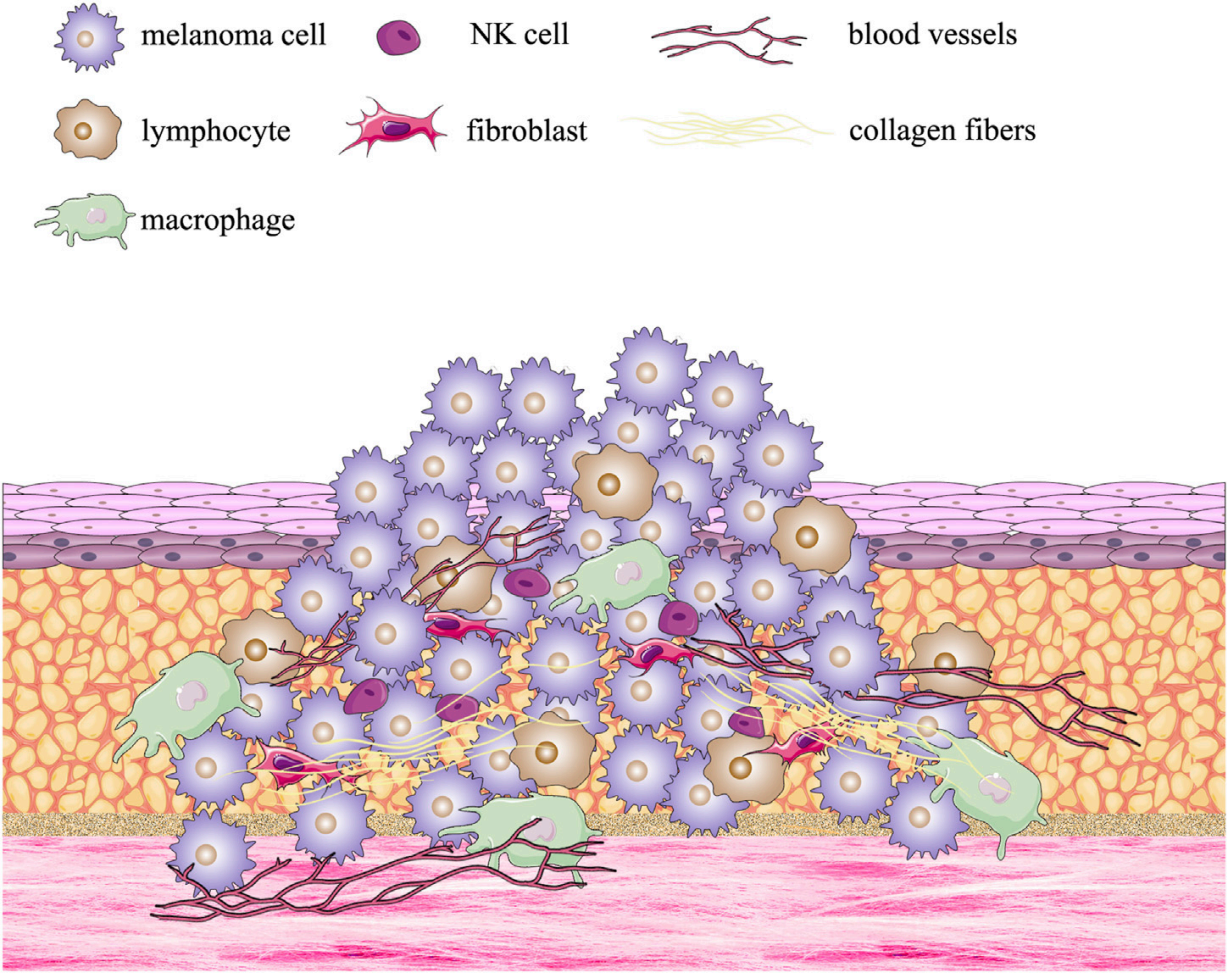
Formation of the multiple melanoma tumor microenvironment.

The patient-derived organoid (PDO) method involves the removal of tissue from a patient’s tumor, its enzymatic treatment, and culture in a scaffold, which leads to the formation of an organoid *in vitro*. Normal cells and their surrounding environment are usually in a dynamic equilibrium, with physiological activities taking place in both. However, a tumor (as a population of malignant cells) disrupts this equilibrium and increases continuously in size and invades its surroundings. The tumor microenvironment (TME), which is conducive to tumor growth, develops as the tumor progresses. TME consists of a complex array of components: blood vessels, immune cells, fibroblasts, bone marrow-derived inflammatory cells, signaling molecules, and extracellular matrix (ECM), and is illustrated in Figure 1. (Sung et al., 2007).

The interaction between immune cells in the TME and the tumor has a crucial role in the proliferation of a tumor, development of drug resistance, and immune evasion (Wu and Dai, 2017). Organoids, by mimicking tumor heterogeneity *in vivo* and preserving TME components (e.g., keratinocytes, cancer-associated fibroblasts) ensure the interaction and dynamic connection between tumor cells and their external environment (Ayuso et al., 2021; Ou et al., 2022). As a result, the dynamic regulation of drugs can be captured, thereby providing insights into the molecular and immune mechanisms of the clinical response to treatment.

PDO models have been utilized for the study of immunotherapy for MM, such as the efficacy of ICI therapy and toxicity of cancer-infiltrating lymphocytes. To establish an effective PDO model, one must explore the interactions and mechanisms between TME components and tumor cells.

Here, we review the correlation (and potential importance) between TME components and PDOs. Also, we demonstrate the advantages of using the PDO model in MM-targeted drug screening and ICI therapy.

## Organoid culture: A new method for immunotherapy research on MM

**FIGURE 2:**
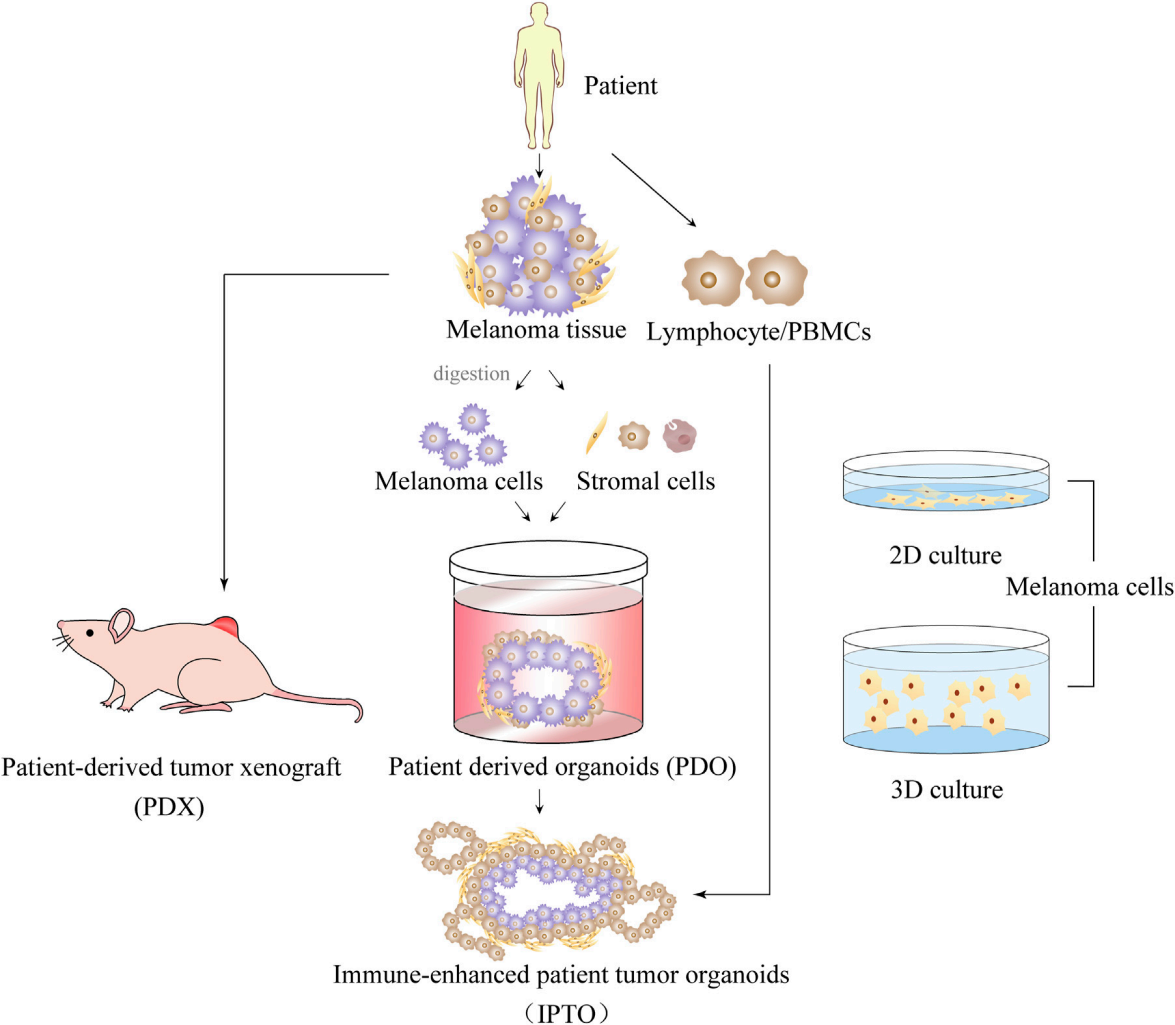
Introduction of 2D and 3D multiple melanoma culture models.

Traditionally, MM has relied on two-dimensional cell culture models. However, this model is limited as it can only culture cells on a flat surface, and is unable to mimic the three-dimensional growth environment and spatial structure of cells, or provide dynamic communication between cells and the external environment, thereby losing the complexity and heterogeneity of cancer. Although high-throughput screening and other studies can be carried out using the two-dimensional cell culture model, it often fails to provide appropriate solutions for problems that require consideration of complex tissue structure and cell-cell interactions. As a result, the predictive correlation between the model and actual clinical outcomes is often poor (Tung et al., 2011; Hickman et al., 2014; Wenzel et al., 2014). To overcome these limitations, high-throughput screening *in vitro* models have been proposed for rapid screening of drugs. There are various models available today for MM studies to choose from, as shown in Figure 2. 3D models have garnered extensive scientific attention over the last two decades because they better represent the structure and function of tumors *in vivo* (Liu et al., 2017).

Among the various 3D culture models, the spheroid model is notable for generating different spheroids with unique characteristics based on the invasive features of the cells cultured. For example, MM cells in advanced stages have a vertical growth phenotype that distinguishes them from early radial growth patterns (Rebecca et al., 2020). Compared to conventional two-dimensional cell cultures consisting of monolayers of cells, these cultures have the ability to form gradients of oxygen, nutrients, metabolites, and soluble signals, thereby giving rise to heterogeneous cell populations. This feature is not achievable in two-dimensional cultures. The use of multicellular spheroid culture in “personalized” drug screening and prediction of clinical treatment for patients with MM holds great potential. However, there are several practical challenges associated with spheroid culture, including the development and maintenance of spheroids of uniform size, the formation of spheroids from a small seed number of cells, the precise control of specific ratios of different cell types in spheroid co-culture, and the lack of reliable, simple, standardized, and high-throughput compatible assays for drug screening using spheroids (Matias et al., 2021). Spheroids present advantages over organoids in terms of preparation and manipulation, as well as faster growth rate, owing to their simpler single or multiple cells composition. Non-etheless, they lack the intricate tissue architecture and cellular interactions of organoids, and fail to mimic the *in vivo* growth environment of cells, which imposes certain limitations.

The patient-derived xenograft (PDX) model involves transplantation of patient-derived tumor tissue into mice for *in vitro* culture. The PDX model ensures tumor heterogeneity and dynamic communication with the host. However, the mouse is deficient in human immune cells, so the full immuno-compatibility is questionable. Furthermore, the expense and long time required for PDX culture presents a hindrance for patients with MM, for whom treatment decisions must be made rapidly.

There is a growing recognition of the importance of using organoids to establish a rapid preclinical model for malignancies (e.g., MM) with the ability to recapitulate the TME. Organoids utilize the selective-differentiation ability of adult cells or stem cells, which are cultured *in vitro* to preserve (as much as possible) the genetic characteristics of the cellular tissue and mimic the true 3D tissue structure *in vivo* (Lancaster and Knoblich, 2014). Compared with previously described 2D or 3D culture models, organoids reproduce the heterogeneity of tumor tissues, but also serve as *in vitro* models to visualize the dynamic and continuous observation of tumors.

**FIGURE 3:**
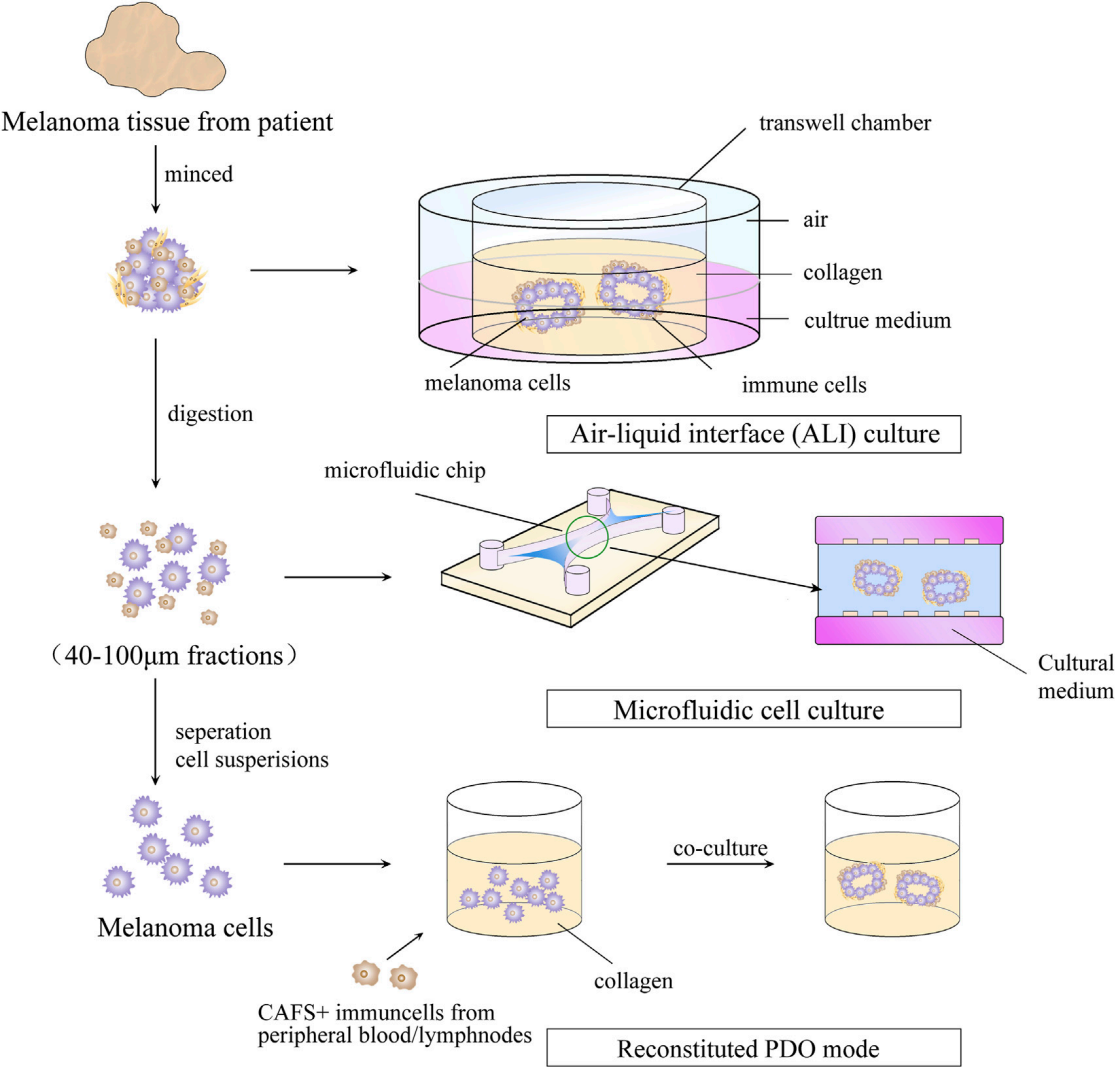
Steps for building a patient -derived organoid model.

The PDO model is an *in vitro* culture system in which tumor-like organs are established using tumor cell tissue collected through resection or core-needle biopsy (Vilgelm et al., 2020). As a result of its high rate of *in vitro* culture and ability to maintain tumor heterogeneity, tumors have been established in various organs, including the colon, prostate gland, breast, lung, and ovary (Gao et al., 2014; Fujii et al., 2016; Sachs et al., 2018; Kim et al., 2019; Kopper et al., 2019). The steps for building PDO model are shown in Figure 3. The obtained tumor tissue must be digested by mechanical or enzymatic means and then cultured. To reproduce the TME, cytokines must be added to promote the development and differentiation of cells and ECM materials that mimic the ECM. Typically, cytokines are used in PDO models as activators or inhibitors of signaling pathways and hormones, and to regulate the proliferation and differentiation of tumor cells. For instance, addition of A83-01 (inhibitor of the transforming growth factor (TGF)-β signaling pathway) is required to prevent tumor cells from undergoing epithelial–mesenchymal transition in the establishment of a prostate-gland organoid (Drost et al., 2016). The role of ECM materials is to simulate the ECM environment and provide a supportive environment for tumor growth. Matrigel™ or basement membrane extract (hydrogel composed of laminin and collagen in proportions similar to those in human basement membrane) are used widely in organoid cultures (Kleinman and Martin, 2005; Heo et al., 2022). Compared with the PDX model, the PDO model offers a simpler and cost-effective alternative, while also reproducing dynamic cell–cell intercellular and cell–ECM connections.

PDOs have a major limitation in that they lack stromal cells and immune cells. To address this issue, Votanopoulos and colleagues developed immune-enhanced organoids (IPDOs) by co-culturing PDOs with peripheral-blood mononuclear cells or the patient’s lymphoid tissue. The resulting IPDO could expand and activate effector T cells, thereby providing a valuable tool for studying the interaction between tumor tissue and the immune system, as well as for drug screening (Troiani et al., 2020; Votanopoulos et al., 2020). Another approach to preserving TME components *in vitro* is to culture digested or minced tumor tissues using microfluidic methods or methods based on the air–liquid interface. This setup creates a symbiotic relationship between tumor cells and immune cells so that the TME-like culture retains the endogenous immune cells and stromal cells. (Neal et al., 2018; Ayuso et al., 2021; Garreta et al., 2021). This means that the PDO model can generate sufficient antigen load to trigger immune responses compared with that in the conventional 3D-culture model of tumor cells. This property lays the foundation for using the PDO model for research on tumor immunology, such as biomarker identification, evaluation of strategies for immune-combination therapy, preparation of tumor-specific T cells, and screening and evaluation of new immunotherapeutic approaches. The recent 5 years of research on MM using organoid technology are summarized in Table 1.

**TABLE 1:** Summary of organoid applications in multiple melanoma.

Tumor models	Cell source	Co-culture components	Matrix	Research content	Author, year
PDO	Melanoma responding to combined TBK1i + anti-PD-1		Matrigel	The innate immune kinase TANK-binding kinase 1 (TBK1) can block ICI responses as an immune evasion gene	Sun et al. (2023)
	Metastatic melanoma with BRAF gene mutation		DMEM	Tandem mutations in BRAF were found to reduce the affinity of Vemurafenib, while inhibition of Notch signaling had a stronger anti-tumor response	Porcelli et al. (2022)
	Melanoma patient	Fibroblast and immune cells	Matrigel	Checkpoint inhibitors and epigenetic modifiers in melanoma results in enhanced anti-tumor function of gammadelta T cells	Ou et al. (2022)
	Melanoma patient		Matrigel\DMEM	A crosstalk between the mitochondrial apoptotic pathway and cell cycle regulation	Bharti et al. (2022)
	Melanoma patient		DMEM	Drug sensitivity testing by tumor organoid and found to be consistent with clinical patient response to treatment	Chen et al. (2022)
	Melanoma patient		Collagen	Breaking peripheral tolerance without regard to antigen specificity by injecting nanoparticles of innate immune stimulants into mice or PDO models	Yin et al. (2021)
	Melanoma patient	PBMCs OR lymph node tissue	Hydrogel	Three-dimensional hybrid immune-enhanced tumor/nodal organs can maintain the immune system and tumor cell viability	Votanopoulos et al. (2020)
	Primary and metastatic melanoma	Autologous lymphoid and myeloid cell populations	Matrigel	Feasibility of FKBP51s as a guide to select and monitor immune checkpoint-targeted therapy in melanoma patients	Troiani et al. (2020)
Air-liquid interface PDO	Melanoma patient	Immune cells	Collagen	Co-culture of PDO with immune cells using the (ALI) method to reproduce the tumor microenvironment and immune activity	Neal et al. (2018)
Organoid-like spheroids	92.1 Mel270 and Mel290 cell line		Collagen	Evaluated the effects of MEKi trametinib alone and in combination with drugs targeting epigenetic regulators	Gonçalves et al. (2019)

Organoid technology has several advantages over other 3D-culture technologies in the context of research on tumor immunology, such as heterogeneity, the ability to achieve massive and stable expansion of cells *in vitro*, the availability of high-throughput assays for drug screening, and the possibility of gene editing. Moreover, organoids can be co-cultured with preserved immune cells or immune cells added exogenously. This strategy allows for preservation of the tumor immune microenvironment (TIME) and detection of the immune system–tumor interaction at different stages of tumor development.

## Immune cells: Key components of the TME

The immune system is a crucial component of the TME and encompasses various cell types, including effector T cells (such as cluster of differentiation (CD)8+ cytotoxic T cells and CD4^+^ effector T cells), natural killer cells, dendritic cells, and M1-polarized macrophages (Hanahan and Weinberg, 2000). The TME influences the immune system by impacting immune cells within the tumor, but also by affecting immune cells recruited to distant sites during tumor invasion. This interaction between the immune system and tumor ultimately affects the growth, invasion, and metastasis of the tumor (Locy et al., 2018). Li and coworkers observed that CD8^+^ T cells in the TME of MM were deregulated, which led to excessive proliferation, cloning, and dynamic differentiation (Li et al., 2019). As a result, immunotherapy with ICIs has gained increasing attention as a means to induce durable clinical responses in patients who have not responded to conventional therapy.

The immune system uses “immune checkpoints” to maintain self-tolerance and suppress immune responses in peripheral tissues, but malignancies can exploit these checkpoints to resist the immune system. For MM, the US Food and Drug Administration has approved use of anti-PD-1 (nivolumab and pembrolizumab), anti-cytotoxic T-lymphocyte associated protein (CTLA)-4 (ipilimumab), and anti-PD-1/anti-CTLA-4 combination therapies (Pardoll, 2012). Recent studies have demonstrated significant pathologic responses and relapse-free survival in patients with stage-III MM treated with anti-CTLA-4 + anti-PD1 monoclonal antibodies, but ∼20% of patients do not respond to this treatment (Kaptein et al., 2022). The efficacy of ICIs in patients with MM is influenced by various factors, including immune cells in the TME, which play a key part (Petitprez et al., 2020).

Myeloid cells are a critical component of the tumor immune system, comprising of tumor-associated macrophages (TAMs) and myeloid-derived suppressor cells (MDSCs) (Solito et al., 2014). TAMs exert their effects on tumor cells through the production of enzymatic activities and cytokines such as interleukin-10 (IL-10) and transforming growth factor b (TGF-b) (Georgoudaki et al., 2016). This cytokine is produced by TAMs to promote tumor growth *in vivo* and stimulate tumor cell invasion *in vitro*, thus acting as a source of resistance to BRAF and MEK inhibitors (Smith et al., 2014). Additionally, inhibiting the expression of TAMs PD-1 has been found to reduce tumor growth by modulating the phagocytic capacity of macrophages, offering new directions for the application of immune checkpoint inhibitor technology in cancer treatment (Gordon et al., 2017).

Additionally, TAMs can influence the tumor stromal environment and promote the growth of solid tumor cells through the secretion of pro-angiogenic factors and secretory matrix metalloproteinases (MMPs) (Fu et al., 2020). TAMs can also influence the invasive and metastatic capacity of tumors, including MM, through various actions. As a result, it is critical to ensure the presence of TAMs as an immune cell component in the PDO model of MM to mimic the heterogeneity of MM tissue structure *in vivo*. Air-liquid interphase (ALI) culture is an organoid culture method that retains native immune and stromal components, allowing immune cells to survive for up to 30 days (Bar-Ephraim et al., 2020). In a recent study using the ALI culture method, TAMs were retained as tumor infiltrating lymphocytes (TILs) components to maintain the integrity of the tumor epithelium and TME. In this study, TILs exhibited activation, expansion and cytotoxic responses to PD-1/PD-L1 checkpoint blockade, successfully mimicking immune checkpoint blockade (Neal et al., 2018). Studying the interaction between MM and TAMs using the PDO model will shed light on the mechanisms of melanoma growth, invasion, and metastasis, and inform the development of new therapeutic strategies.

Despite the capability of utilizing PDOs for drug screening and immunotherapy assessment, the intricate relationship between the immune system and tumor is not known. This knowledge gap is largely due to the inability of the reconstructed TME organoid culture model to preserve the original characteristics of the TIME because post-digestion screening often results in the removal of immune cells. Currently, the function of immune cells in the TME is often explored through co-culture of patient-derived tumor cells and immune cells added exogenously to form organoids. Studies have demonstrated that ICI application in PDO models of MM leads to an increase in infiltration by CD8^+^ T cells and the toxicity of γδ T cells, which is often indicative of a favorable prognosis for the patient. This phenomenon, in turn, results in the apoptosis of tumor cells in melanoma PDOs (Gao et al., 2007; Pagès et al., 2018; Ou et al., 2022).

For patients with advanced MM in whom conventional therapies are not efficacious, a combination of ICIs with anti-CTLA4 + anti-PD-1 can be used. When this combination was administered to an *in vitro* model of the tumor and interleukin (IL)-2 was added, an expansion in the number of tumor-specific CD8^+^ T cells and an improvement in the multifunctionality of pro-inflammatory cytokines were observed. This final triple therapy, compared with anti CTLA4 + anti PD1 therapy alone, elicited better therapeutic outcomes (Kaptein et al., 2022). One study utilized MM cells and lymph-node cells from the same patient to co-culture immune-enhanced patient tumor organoids (iPTOs). Immunotherapy tests showed that 85% of iPTO responses were consistent with the clinical responses of the samples. In another trial, peripheral T cells were transferred into the patient’s PTO after circulation through the iPTO, which resulted in tumor killing and suggested the potential of iPTO to generate adaptive immunity (Votanopoulos et al., 2020). The co-culture of MM-like organs with autologous lymphocytes also revealed that the FKBP51 immunophilin proteins expressed in immune cells could guide the selection and monitoring of ICI therapy in patients with MM (Troiani et al., 2020). Regulatory T cells (T_regs_) are, in general, associated with a worse prognosis in cancer therapy (Takeuchi and Nishikawa, 2016). However, recent research suggests that T_reg_ populations may be positively associated with the efficacy of therapy against CTLA-4 within ICI treatment (Hamid et al., 2011; Arce Vargas et al., 2018). Further studies are needed to decipher the dynamic interactions between the tumor, host, and immune cells to understand the regulatory mechanisms of sensitivity and resistance to immunotherapy.

To this end, patient-derived 3D tumor explants (PDEs) have emerged as a promising preclinical cancer model. Unlike classic models, PDEs are cultured from freshly resected cancer tissue without deconstruction or reconstruction. Hence, the histological features of the tumor (including stromal cells and immune cells) are retained. This unique property allows PDEs to recapitulate tumor heterogeneity and the role of immune cells in the TME, making it possible to predict drug responses *in vivo* or assess the effect of targeted drugs (Shafi et al., 2018; Collins et al., 2020; Powley et al., 2020).

## Cancer-associated stromal cells and normal cells

As one of the most important components of the TME, cancer-associated stromal cells are composed of cancer-associated fibroblasts (CAFS), endothelial cells, and adipocytes (Giraldo et al., 2019). CAFs are activated by cancer cells in tumor tissue and possess the properties of myofibroblasts, having a crucial role in supporting the growth and regulating the development of tumor cells (Kobayashi et al., 2019). With the help of secreted cytokines, chemokines, growth factors, and intercellular signaling, most CAFs promote the development of cancer cells, particularly in terms of proliferation and evasion from the immune system (Farhood et al., 2019). However, some CAFs exhibit tumor-suppressive properties, and are known as cancer-suppressive cancer-associated fibroblasts. Mizutaniet al. found that meflin (a glycosylphosphatidylinositol-anchored protein expressed on CAFs-derived cells) inhibited the growth and development of tumor cells by blocking the differentiation of tumor cells (Mizutani et al., 2019).

There is a complex interaction between CAFs and immune cells in the TME, including involvement in the regulation of specific and non-specific immunity. For example, CAFs can exert immunosuppressive effects through IL-6, CXC chemokine ligand (CXCL)9, and TGF-β, while producing IL-1β, IL-8, IL-10, tumor necrosis factor-α, monocyte chemotactic protein-1/CCL2, CXCL12, and β-type interferon to regulate immune cells directly (Fearon, 2014). Moreover, MM induces the migration and conversion of normal fibroblasts to CAFs through paracrine secretion (Flach et al., 2011). *In vivo* and *ex vivo* models have shown that CAFs contribute to the growth, invasion, and drug resistance of MM cells (Kääriäinen et al., 2006; Gaggioli et al., 2007; Zhou et al., 2015; Dvořánková et al., 2017). CAFs can also influence the progression of melanoma by interacting with immune cells, for example, by forming inflammatory niches with TAMs and promoting the development of melanoma drug resistance (Young et al., 2017). Normal fibroblasts and cortical cells from normal tissues also play a part in regulating MM cells in the TME.

Studies targeting normal cells in co-culture-like organs are slowly revealing the role of normal cells in promoting or inhibiting tumor development. In normal tissues, a functional symbiosis between melanocytes and keratinocytes is necessary for homeostasis. However, if MM is cultured in single cells, the addition of exogenous keratinocytes to the co-culture reveals a phenotype more similar to that observed *in vivo* (Beaumont et al., 2013). Disruptions in the normal regulation of keratinocytes (e.g., E-cadherin-mediated regulation of keratin-forming cells) can contribute to the production of MM cells (Hsu et al., 2000). Ayuso et al. (2021) co-cultured MM cells with fibroblasts and keratinocytes using microfluidic methods. They found that the presence of both cell types resulted in the production of chemokines (including IL-6, IL-8, IL-1β, IL-1α) and reduced the redox rate of MM cells in a 3D environment. Those data suggested that fibroblasts and stromal cells have roles in promoting MM development. The presence of fibroblasts also resulted in significant suppression of infiltration by immune (e.g., γδ T) cells in 3D models of MM co-cultured with them, including patient-derived MM-like organs, which reduced the toxicity of tumor-infiltrating lymphocytes to the tumor (Ou et al., 2022). The complex interplay between MM cells and stromal cells is mediated by various factors from cells, and this is a new approach in studying the mechanisms of MM development through the PDO model. Simultaneously, deeper understanding of the interactions between stromal cells and immune cells in the TME may aid discovery of new targets for immunotherapy for MM cells.

## Summary and outlook

To simulate the characteristics of MM in the body effectively, the MM model must be improved continually. Among the various 2D and 3D culture models, organoids possess unique advantages, such as a high success rate, stable transmission, rapid proliferation, and high heterogeneity. The development of technologies based on high-throughput screening (e.g., proteomics and genomics) has ensured that organoid-culture technology is an effective tool for studying MM biology but also an extremely valuable method for drug screening and immunotherapy research (Fang and Eglen, 2017). In particular, the PDO model has unique advantages for immunotherapy research (e.g., ICI therapy) because it preserves the components and heterogeneity of tumors while simultaneously reproducing the TME and ensuring communication between tumor cells and their environment. Study of the progression, invasion, drug resistance, and immune-evasion mechanisms of MM using PDO is crucial for evaluating anti-tumor therapies, especially immunotherapy (Boucherit et al., 2020).

However, the classic PDO model faces the challenge of a lack of immune cells. To observe the dynamic interaction between the immune system and MM, immune cells must be added exogenously and co-cultured, but they eventually become exhausted. TME organoid models based on microfluidic technology can preserve immune cells and stromal cells, but the next challenge is to improve the success rate and ensure the stability of cultures (Yuki et al., 2020). The *in vitro* 3D culture technology PDE, while preserving the complete composition and structure of patient tissue, is limited in its development due to difficulty in conducting high-throughput drug screening and relatively short window for operation (Powley et al., 2020; Rodolfo et al., 2022). Formation of the tumor microvasculature and distribution of oxygen and nutrients from MM to other organs are also crucial for reproducing the TME in the body (Rodolfo et al., 2022). The development of non-invasive methods (e.g., liquid biopsy) will further advance organoids as a platform for the prediction and drug screening of personalized treatment for patients (Maru et al., 2019).
